# Biobanking of Fresh-Frozen Human Adenocarcinomatous and Normal Colon Tissues: Which Parameters Influence RNA Quality?

**DOI:** 10.1371/journal.pone.0154326

**Published:** 2016-04-28

**Authors:** Thibaut Galissier, Christophe Schneider, Saviz Nasri, Lukshe Kanagaratnam, Caroline Fichel, Christelle Coquelet, Marie-Danièle Diebold, Reza Kianmanesh, Georges Bellon, Stéphane Dedieu, Aude Marchal Bressenot, Camille Boulagnon-Rombi

**Affiliations:** 1 Laboratoire d’Anatomie Pathologique, Centre Hospitalier Universitaire, Reims, France; 2 CNRS UMR 7369, Matrice Extracellulaire et Dynamique Cellulaire, MEDyC, Reims, France; 3 Laboratoire SiRMa, Université de Reims Champagne-Ardenne, UFR Sciences Exactes et Naturelles, Reims, France; 4 Tumorothèque de Champagne Ardenne, Reims, France; 5 Unité d’Aide Méthodologique, Centre Hospitalier Universitaire, Reims, France; 6 Laboratoire d’Anatomie Pathologique, Université de Reims Champagne-Ardenne, UFR Médecine, Reims, France; 7 Service de Chirurgie Digestive, Centre Hospitalier Universitaire, Reims, France; 8 Laboratoire de Biochimie, Centre Hospitalier Universitaire, Reims, France; Centro Nacional de Investigaciones Oncológicas (CNIO), SPAIN

## Abstract

Medical research projects become increasingly dependent on biobanked tissue of high quality because the reliability of gene expression is affected by the quality of extracted RNA. Hence, the present study aimed to determine if clinical, surgical, histological, and molecular parameters influence RNA quality of normal and tumoral frozen colonic tissues. RNA Quality Index (RQI) was evaluated on 241 adenocarcinomas and 115 matched normal frozen colon tissues collected between October 2006 and December 2012. RQI results were compared to patients’ age and sex, tumor site, kind of surgery, anastomosis failure, adenocarcinoma type and grade, tumor cell percentage, necrosis extent, HIF-1α and cleaved caspase-3 immunohistochemistry, and *BRAF*, *KRAS* and microsatellites status. The RQI was significantly higher in colon cancer tissue than in matched normal tissue. RQI from left-sided colonic cancers was significantly higher than RQI from right-sided cancers. The RNA quality was not affected by ischemia and storage duration. According to histological control, 7.9% of the samples were unsatisfactory because of inadequate sampling. Biobanked tumoral tissues with RQI ≥5 had lower malignant cells to stromal cells ratio than samples with RQI <5 (p <0.05). Cellularity, necrosis extent and mucinous component did not influence RQI results. Cleaved caspase-3 and HIF-1α immunolabelling were not correlated to RQI. *BRAF*, *KRAS* and microsatellites molecular status did not influence RNA quality. Multivariate analysis revealed that the tumor location, the surgical approach (laparoscopy versus open colectomy) and the occurrence of anastomotic leakage were the only parameters influencing significantly RQI results of tumor samples. We failed to identify parameter influencing RQI of normal colon samples. These data suggest that RNA quality of colonic adenocarcinoma biospecimens is determined by clinical and surgical parameters. More attention should be paid during the biobanking procedure of right-sided colon cancer or laparoscopic colectomy specimen. Histological quality control remains essential to control sampling accuracy.

## Introduction

Despite advances in screening, diagnosis, and treatment, colorectal cancer (CRC) is the third most common cancer and the fourth-leading cause of cancer death worldwide [1,2]. Pathological staging is the only prognostic classification used in clinical practice to select patients for adjuvant chemotherapy [3]. However, the histoclinical parameters apprehend only poorly the heterogeneity of disease and are insufficient for recurrence and prognostic prediction in an individual patient [4]. Genetic and genomic analyses of colorectal adenocarcinomas uncovered a number of germline and somatic keys mutations that drive tumorigenesis at a molecular level along the tumor progression [5–7]. Among the molecular markers that have been extensively investigated for CRC characterization and prognosis, microsatellite instability (MSI) is the only marker that was reproducibly found to be a significant prognostic factor in CRC [8,9]. Molecular research on CRC aims to identify markers involved in tumor progression, leading to a better understanding of the carcinogenesis process, discovering new prognostic markers and novel therapeutic targets. The gene expression profiling analysis with microarray technology shows great potential in cancer research and medical oncology, mapping simultaneously the expression of thousands of genes in a single tumor sample and giving a measurement of articulated genes expression patterns [10,11]. This type of study requires high quality biospecimens. Thus, fresh-frozen tissue is the favored biospecimen because it produces a high yield and high quality of nucleic acids compared to formalin-fixed paraffin-embedded (FFPE) tissue biospecimen in which nucleic acids are fragmented [12,13]. Moreover validated protocols for genomic and expression profiles studies on FFPE material are not yet available [14,15].

A human tissue Biobank is a biorepository that accepts, processes, stores and distributes biospecimens. It associates data for use in research and clinical care [16]. Biobanks can provide researchers a reliable and organised source of human tissue for RNA-based analysis. RNA integrity is of utmost importance when applications involve RNA quantification for gene expression studies such as RT-PCR and cDNA microarray. RNA is highly susceptible to degradation, thus careful and standardized handling procedure to preserve RNA quality of the biospecimens is needed in tissue bank [17,18]. RNA integrity indicators such as the RNA quality index (RQI) are commonly used in Biobanks as an objective and reproducible RNA quality control [17,19]. The RQI score is similar to another widely used RNA quality indicator: the RNA Integrity Number (RIN) [20–22]. The RQI and RIN are numerical representation of electrophoretic measurements of the whole RNA integrity. These numerical scales are comprised between the most degraded RNA score: 1 and the most intact RNA score: 10. In several studies, RNA with a RQI/RIN <5 were considered as not reliable for further analyses, whereas gene expression analysis needs imperatively RNA with a RQI/RIN >7 [18,22]. Using degraded RNA leads to incorrect and hardly reproducible quantification results, both in microarray experiments and real-time PCR. Lack of reproducibility in various studies raises concern about variation in quality of tissues used for such studies [23,24].

In recent years, considerable attention was given to the influence of preanalytic effects on biospecimen quality. The preanalytical phase summarizes all procedures at various stages–from tissue sampling to gene expression analysis–that may potentially affect the quality of the sample hence the experimental results and their reproducibility [23,25–28]. As a consequence it is now recommended that researchers using biospecimens ensure that key preanalytical variables (BRISQ Tier 1 items) from the Biospecimen Reporting for Improved Study Quality (BRISQ) guidelines are available [23,27]. Knowledge of these key variables can improve reproducibility and homogeneity of studies using biospecimens. However, the real impact of some parameters on RNA quality such as: the kind of surgery, cold ischemia duration, storage temperature and duration, is actually unknown or controversial.

Histological control of stored tissue is also a crucial step prior to embarking on time-consuming, labor-intensive, and costly projects. Approximately 10% of the frozen samples are unsuitable for a molecular analysis mainly because of tissue sampling inadequacy (no tumor or insufficient quantity of malignant cells) [29]. According to the literature, the most important parameters controlled by the histological analyses are tumor cells amount and necrosis extent [28,29]. However, the relationship between histological parameters such as tumor type, malignant/stromal cells percentage, type of stromal cells, necrosis extent and the RNA quality remains unknown. Moreover, immunohistochemical markers canditates to be related to RNA quality are not yet studied.

According to the literature, RNA integrity from gastrointestinal tract tissues and particularly colonic tissue is of significantly lower quality compared to other organs [17,26]. Therefore identification of parameters that could influence RNA quality seems essential to improve procedures ensuring the best RNA quality of such biospecimens.

The aim of this study was to search among clinical, histopathological, and molecular factors those influencing RNA quality of adenocarcinomatous and matched normal colon tissues.

## Materials and Methods

### Population studied

The study was conducted on tumoral and non tumoral colonic tissues sampled on colectomies received at the Pathology Department of Reims University Hospital (France) for histological diagnosis from October 2006 to December 2012 and stored in the Tumorothèque de Champagne Ardenne Biobank. The patients had not been treated with chemotherapy or radiotherapy before surgery. The study was performed in accordance with the ethical standards laid down in the Declaration of Helsinki. Written patients’ consent for biobanking and biospecimen use was obtained in all cases. Approval had been obtained from the Tissue Bank Management Board (Autorization number DC-2008-374) for the study.

Clinical data and preanalytical variables including age at surgery, sex, tumor site, cold ischemia time, and *KRAS*, *BRAF*, MSI molecular status were obtained from the Tumorothèque de Champagne Ardenne Biobank database and the patients’ medical records. Tissue cold ischemia time referred to the time from tissue excision to snap-freezing [27].

A total of 241 colonic adenocarcinoma tissue samples and 115 matched normal colonic mucosa samples were available for the study. The patients were 106 females (44%) and 135 males (56%). The mean age at time of resection was 70 years ±11 and median 71 years [43–90 years]. The adenocarcinoma was located in the right colon in 104 cases and in the left colon in 137 cases. Population data are summarized in Table 1.

**Table 1:** Clinical, pathological and molecular available data.

Variables	n	n (%) or mean ±SD
**Sex**	241	
female		106 (44.0)
male		135 (56.0)
**Age (years)**	241	70.1 ±11.2
**Location**	241	
right		104 (43.2)
left		137 (56.8)
**Kind of surgery**	239	
Right hemicolectomy		104 (43.5)
Transverse colectomy		10 (4.2)
Left colectomy		35 (14.6)
Sigmoidectomy		29 (12.2)
High anterior resection		55 (23)
Total colectomy		6 (2.5)
**Surgical approach**	222	
Laparoscopic colectomy		10 (4.5%)
Open colectomy		212 (95.5%)
**Anastomotic leakage**	240	
Yes		16 (6.7%)
No		224 (93.3%)
**Surgery duration (minutes)**	16	132.5 ±82.01
**Ischemia duration (minutes)**	181	70.2 ±50.9
**Tumor grade**	237	
1		20 (8.4)
2		201 (84.8)
3		16 (6.8)
**MSI status**	141	
MSS		119 (84.40)
MSI		22 (15.60)
**BRAF**	94	
wt		79 (84.0)
mutated		15 (16.0)
**KRAS**	90	
wt		53 (58.9)
mutated		37 (41.1)

MSI: Microsatellite instable, MSS: Microsatellite stable, wt: wild type.

The tumor grade was established according to the WHO (1) independently to MSI status (1 = well; 2 = moderately; 3 = poorly).

#### Tissue Banking Process

At the time of the surgical resection, the specimens were transported at room temperature to the Pathology Department, where time of arrival was recorded. Colonic tumor tissue samples (TT) and matched normal colon tissues (NT) were collected as quickly as possible. A sample was excised from the tumor with a scalpel blade and then divided into two halves: one for biobanking and one for formalin fixation and mirror histological control [30–32]. The same process was realized on normal colonic mucosa distant from the tumor. Immediately after removal, tumor and normal tissue samples were stored in labeled cryovials, snap-freezed in liquid nitrogen, and then transferred to an ultra low-temperature refrigerator (-80°C) for storage. The tumor and normal tissue mirror control specimens were formalin fixed and paraffin-embedded (FFPE) and then underwent routine histopathological processing. Corresponding slides and paraffin blocks were stored at room temperature.

### Histopathology

Hematoxylin-phloxine-saffron (HPS) slides were performed on 4-μm-thick tissue sections of all the NT and TT FFPE mirror blocks. Histological evaluations were performed by two pathologists (T.G. and C.B-R.) blinded to the RQI results. In case of discrepancies a consensus diagnosis was reached. The adenocarcinoma subtypes, and grade were evaluated according to The World Health Organization (WHO) criteria [1]. The following criteria were evaluated on TT samples: the ratio of malignant to stromal cells, the cellularity, the necrosis extent, the presence and percentage of ulceration, the presence and percentage of mucinous component and the contamination by normal residual tissue. Among the stromal component, the percentages of stromal inflammatory cells, endothelial cells and fibroblasts were separately assessed. The percentages were calculated as the ratio of each stromal cell type to all stromal cells.

**Fig 1:**
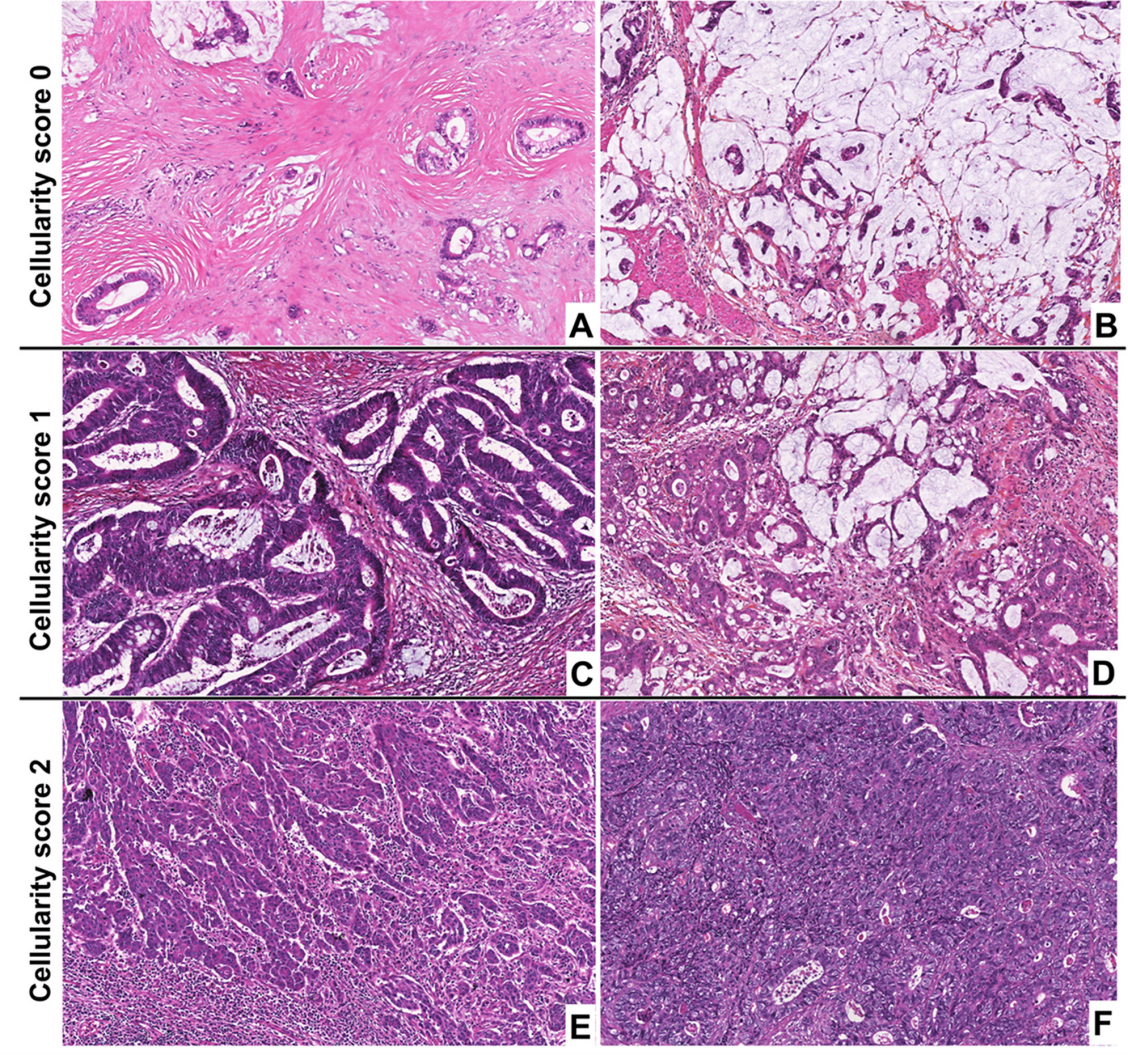
Illustration of the cellularity score, (Hematein-Phloxin-Saffron, x10). **A-B.** Cellularity score 0 in two adenocarcinomas with low cell density. **C-D:** Cellularity score 1 in two adenocarcinomas with moderate cell density. **E-F:** Cellularity score 2 in two adenocarcinomas with high cell density.

The TT cellularity was a semi-quantitative evaluation of the global cellular density of the whole sample: malignant and non-malignant cells, scored as follow: 0 = low, 1 = moderate and 2: high. Examples are given in Fig 1.

### Immunohistochemistry

We aimed to evaluate if immunohistochemical expression of apoptosis or ischemia markers (cleaved caspase 3 and HIF-1α respectively) were correlated to RNA quality. Thus, these markers could be used on mirror samples as a quality control. Immunostaining was performed on 4-μm-thick sections with anti-Cleaved Caspase-3 (rabbit polyclonal, 1:600 Cell Signaling Technology, Danvers, MA, USA), and anti-Human HIF-1α (mouse monoclonal, clone 54/HIF-1α, 1:100 BD biosciences, San Jose, CA, USA) primary antibodies on the BenchMark XT (Ventana Medical System, Tucson, AZ, USA) automated staining instrument. Briefly, after deparaffinization, the tissue sections were incubated with the Cell Conditioner 1 (EDTA, pH 8.4) for 64 min, followed by preprimary peroxidase inhibition and incubation with the primary antibody at 37° for 32 min. Then, the staining reaction was performed by using the ultraView Universal DAB v3 Kit (Ventana Medical System). The counterstain and post-counterstain comprised Haematoxylin and bluing reagent.

Cleaved caspase 3 and HIF-1α immunohistochemistry were assessed separately by determination of the percentages of positive tumor cells and stromal cells. The percentages of stromal or tumor cells were calculated as the number of positive stromal or tumor cell divided by the number of total stromal or tumor cells respectively.

### RNA extraction

The frozen sample was weighted just before RNA extraction process. RNA extraction and purification was performed on the whole frozen tissue sample after 24 to 48 hours homogenization and lysis. The Maxwell® 16 LEV simply RNA tissue kit (Promega, Madison, USA), a paramagnetic bead-based RNA purification, with enzymatic DNA elimination, was used according to the manufacturer’s instructions on the Promega‘s robotics platform: the Maxwell® 16 Research Instrument (Promega). RNA was eluted in a volume of 50 μL. The concentration of total RNA (ng/ μL) was determined by using a Picodrop uL spectrophotometer (Picodrop, Hinxton, United Kingdom).

### Determination of RNA quality

RNA quality index (RQI) was determined using the Experion ™ automated electrophoresis system (Bio-Rad, Marnes-la-Coquette, France), a microfluidics-based system electrophoresis, according to the manufacturer protocol. The RQI software algorithm allows for the classification of total RNA, based on a numbering system from 1 to 10 (1 being the most degraded and 10 being the most intact). The RQI calculations take into account 3 electrophoregram regions: the 28S, the 18S and the pre-18S regions [20]. The RQI results are similar to the RNA Integrity Number results, another microfluidics-based system electrophoresis that analyses RNA integrity [20,21].

RQI values were classified as “of sufficient quality” (RQI ≥5) or as “inadequate for further analyses” (RQI<5) according to the literature [17,19,30,31].

### 5’3’ mRNA ratio integrity assay

The principle of this qPCR-based assay is based on the fact that anchored oligo-dT primed reverse transcription proceeds from the 3’ poly-A tail to the 5’ start, being interrupted if mRNA is fragmented due to degradation. Two qPCR assays targeting either the 3’ end or the 5’ start of 2 commonly used housekeeping genes: TATA-Binding Protein (*TBP*) and Beta-2-microglobulin (*B2M*) were evaluated. Total RNA (250 ng) was reverse-transcribed using VERSO cDNA kit (Thermo Fisher Scientific, Waltham, MA, USA) according to the manufacturer’s instructions using oligodT primers. Primers were designed according to human sequences encoding for *TBP* and *B2M*. The sequences of the pairs of primers used were: *3’TBP* (5’-CAGCGTGACTGTGAGTTGCT-3’ and 5’-AACTGTGGTTCATGGGGAAA-3’), *5’TBP* (5’-CGGCTGTTTAACTTCGCTTC-3’ and 5’-CTGAATAGGCTGTGGGGTCA-3’), *3’B2M* (5’-TGCTTAGAATTTGGGGGAAA-3’ and 5’-CCAGATTAACCACAACCATGC-3’), *5’B2M* (5’-TATCCAGCGTACTCCAAAGA-3’ and 5’-GACAAGTCTGAATGCTCCAC-3’). 5’ and 3’ Ct values were determined and the difference in Ct value between both assays was calculated and defined as the 5‘3’ dCt ratio. The 5’3’ dCt is inversely correlated to RNA quality. All primers were synthesized by Eurogentec (Eurogenetec, Liège, Belgium). Real-time PCR was performed using an Absolute SYBR Green Rox mix (Thermo Fisher Scientific), on a CFX 96 real time PCR detection system (Bio-Rad). PCR conditions were 15 min at 95°C, followed by 40 cycles each consisting of 15 s at 95°C (denaturation) and 1 min at 60°C (annealing/extension). The specificity of PCR amplification was checked using a heat dissociation curve from 65°C to 95°C following the final cycle. The cycle threshold (Ct) values were recorded with Bio-Rad CFX Manager^™^ 3.0 software (Bio-Rad).

### Molecular analysis

Tumor DNA was extracted from formalin-fixed, paraffin-embedded (FFPE) tissues after macrodissection of a preselected area with the highest adenocarcinomatous cells density. Each sample analyzed contained at least 30% of tumor cells. DNA was extracted on Maxwell® 16 Instruments automaton after tumor macrodissection with Maxwell® 16 FFPE Tissue LEV DNA Purification Kit (all products from Promega Corporation, Madison, USA).

*BRAF*^V600E^ mutation detection was assessed by allelic discrimination using TaqMan probes as previously described [32]. *KRAS* gene mutations on codons 12 and 13 were searched by pyrosequencing analysis using the Pyromark Q96 kit (Quiagen GmbH, Hilden, Germany) as previously described [32]. The MSI status was determined by Multiplex PCR analysis of 5 mononucleotitic markers (BAT25, BAT26, MONO27, NR21, NR24) and 2 pentanucleotidic markers (PentaC and Penta D) using the MSI Analysis System kit, Version 1.2 (Promega Corporation, Madison, USA) as previously described [32].

### Statistical Analysis

Non tumoral and tumoral colon tissues RQI were compared by paired T-test. Quantitative variables were compared between RQI ≥5 and RQI<5 groups using the Student test. Indeed, RNA with RQI<5 were considered as not reliable for further analyses such as microarray analyses and qRT-PCR of long amplicons (>200bp) whereas RNA with a RQI at least >5 is suitable for such analyses [17,19,30,31]. Qualitative variables were compared by Fisher’s exact test or Khi-2 test, as appropriate. We used a linear regression technique for cycle threshold evaluation compared to RQI values. Multivariable analysis by logistic regression with backward elimination steps was performed including all variables that had a p value <0.20 by univariate analysis. Statistical analyses were performed with SAS version 8.2 (SAS institute Inc, Cary, North California). For all tests, *p*< 0.05 were considered to be statistically significant.

## Results

### Comparison between non tumoral and tumoral colon tissue RQI

The RQI was successfully determined in all the 241 TT samples and 115 NT samples. The RNA was considered as “of sufficient quality” (RQI ≥5) in 56 of the 115 NT samples (48.7%) and 188 of the 241 TT samples (78%). The mean RQI of the NT tissue sample was 4.8 ±2 and median 4.25 [1.9–8.8]. The RQI was significantly higher (p <0.0001) in TT samples with a mean of 6.5 ±1.9 and median of 7.3 [1.9–10]. RQI values were 1.7 higher in TT than in paired NT samples.

### Parameters influencing RQI of non tumoral colon tissues

There was no significant difference with the clinical parameters: age, sex and anatomical site between the RQI <5 and ≥5 groups. The kind of surgery, the surgical approach, the cold ischemia duration, the sample weight, and the storage duration were not statistically different between the two groups. Surgery duration was available in only 9 cases. Spearman’s correlation coefficient ρ between RQI values and surgery duration was 0.36 (weak). Histological examination confirmed that all samples were normal colonic mucosa without any contamination by adenoma or adenocarcinoma tissue. There was no statistically significant difference of cleaved caspase 3 and HIF-1α immunohistochemical expression between the 2 groups. These results are summarized in Table 2.

**Table 2:** Parameters associated with RNA quality in non tumoral colonic tissue.

	n	RQI < 5n (%) or mean ±SD	RQI ≥ 5n (%) or mean ±SD	p-value
**Sex n (%)**	115			
female		25/59 (42.4)	28/56 (50)	0.41 ‡
male		34/59 (57.6)	28/56 (50)	
**Age mean (years)**	115	68.5 ±9.8	68.1 ±12	0.81 †
**Location n (%)**	115			
Right		29/59 (49.2)	22/56 (39.3)	0.29 ‡
left		30/59 (50.8)	34/56 (60.7)	
**Surgical approach**	106			
Laparoscopic colectomy		2/3 (3.6)	1/3 (2)	
Open colectomy		53/103 (96.4)	50/103 (98)	0.62 ‡
**Anastomotic leakage (Yes/Total)**	114	7/59 (11.9)	3/55 (5.4)	0.32 ∫
**Cold ischemia duration (minutes)**	89	67.8 ±53.9	60 ±48.1	0.48 †
**Storage duration (years)**	115	6.2 ±1.9	6.9 ±1.8	0.06 †
**NT weight mean (grams)**	113	0.16 ±0.08	0.17 ±0.07	0.63 †
**NT caspase mean (%)**	113	0.5 ±1	0.6 ±1.5	0.73 †
**NT HIF-1α mean (%)**	113	2 ±6	3 ±8	0.34 †

NT: normal tissue, RQI: RNA Quality Index, SD: Standard Deviation

‡: Khi2 test

†: T-test

∫: Fisher’s exact test.

### Parameters influencing RQI of tumoral colon tissues

Results of clinical, histological and molecular parameters association with RQI are summarized in Table 3.

**Table 3:** Parameters associated with RNA quality in tumoral tissue.

	n	RQI < 5n (%) or mean ±SD	RQI ≥ 5n (%) or mean ±SD	p-value
**Sex**	241			0.14 ‡
female		28/53 (52.8)	78/188 (41.5)	
male		25/53 (47.2)	110/188 (58.5)	
**Age (years)**	241	70.7 ±9.9	70 ±11.6	0.68 †
**Location**	241			**0.01** ‡ *
right		31/53 (58.5)	73/188 (38.8)	
left		22/53 (41.5)	115/188 (61.2)	
**Surgical approach**	222			0.01 ∫*
Laparoscopic colectomy		6 (12)	4 (2.3)	
Open colectomy		44 (88)	168 (97.7)	
**Anastomotic leakage (Yes/Total)**	240	6/53 (11.3)	10/187 (5.3)	0.13 **∫**
**Cold ischemia duration (minutes)**	181	59.9 ±44.4	73.6 ±52.5	0.12 †
**Storage duration (years)**	241	5.8 ±1.9	5.9 ±1.8	0.76 †
**Sample weight (grams)**	216	0.18 ±0.12	0.19 ±0.07	0.53 †
**Type**	237			0.23 ‡
Non mucinous adenocarcinoma		42/52 (80.8)	162/185 (87.6)	
Mucinous adenocarcinoma		10/52 (19.2)	23/185 (12.4)	
**Mucinous component percentage**	233	7.2 ±20.8	6.3 ±20.1	0.77 †
**Grade**	237			0.09 **∫**
1		3/52 (5.8)	17/185 (9.2)	
2		42/52 (80.8)	159/185 (85.9)	
3		7/52 (13.4)	9/185 (4.9)	
**Ulceration**	237			0.06 ‡
absent		50/52 (96)	161/185 (87)	
present		2/52 (4)	24/185 (13)	
**Ulceration percentage**	233	3.2 ±8.2	3.5 ±8.1	0.77 †
**Cellularity**	237			0.2 ‡
0		16/52 (30.8)	38/185 (20.6)	
1		21/52 (40.4)	104/185 (56.2)	
2		15/52 (28.8)	43/185 (23.2)	
**Malignant/stromal cells (%)**	237	44.2 /55.8	35.5 /64.5	**0.02** † *
**Necrosis (%)**	237	5.1 ±13.9	3.6 ±9	0.44 †
**Caspase (%)**	235			
Tumor cells		4.7 ±4.1	3.6 ±3.5	0.07 †
Stromal cells		0.2 ±0.5	0.1 ±0.6	0.71 †
**TT HIF-1 α (%)**	235			
Tumor cells		8.5 ±12.3	9.8 ±13.9	0.55 †
Stromal cells		7.9 ±17.4	4.6 ±9.7	0.20 †
**MSI**	141			0.78 **∫**
MSS		25/30 (83.3)	94/111 (84.7)	
MSI		5/30 (16.7)	17/111 (15.3)	
**BRAF**	94			0.33 **∫**
wt		17/22 (77.3)	62/72 (86.1)	
mutated		5/22 (27.7)	10/72 (13.9)	
**KRAS**	90			0.49 ‡
wt		11/21 (52.4)	42/69 (60.9)	
mutated		10/21 (47.6)	27/69 (39.1)	

‡: Khi2 test

†: T-test

∫: Fisher’s exact test

SD: Standard Deviation, TT: tumoral tissue, wt: wild type

* Significant *p*-value.

Cellularity was evaluated semi-quantitatively for NT and TT as follow: 0 = low; 1 = moderate; 2 = high. The tumor grade was established according to the WHO (1) independently to MSI status (1 = well; 2 = moderately; 3 = poorly).

#### Clinical and surgical parameters

There was no significant difference with the clinical parameters: age and sex between the RQI <5 and ≥5 groups. RQI values were significantly (p = 0.05) higher in left-sided colon adenocarcinomas (mean 6.7 ±1.8; median 7.3 [1.9–10]) than in right-sided colon adenocarcinomas (mean 6.2 ±2; median 7 [1.9–9.2]). Significantly more left-sided TT samples had a RQI ≥5 (115/137, 83.9%) than right-sided TT (73/104, 70.2%) (p = 0.01). The surgical approach but not the type of surgery influenced RQI. The RQI results were lower in laparoscopic colectomy specimens than in open colectomy specimens. Moreover, RQI results were lower in case of anastomotic leakage. Surgery duration was available in only 16 cases. Spearman’s correlation coefficient ρ between RQI values and surgery duration was 0.27 (weak).

Multivariate analyses using all the potential influencing variables with p<0.20, revealed that 3 clinical/surgical parameters significantly influence RQI results: the tumor location, the surgical approach (open *versus* laparoscopic colectomy) and the occurrence of anastomosis leakage. Results of the multivariate analyses are summarized in Table 4.

**Table 4:** Parameters associated with RNA quality in tumoral tissue on multivariate analysis.

Variables	Odds ratio (95% CI)
TT cellularity 1 *vs*. 0	0.83 (0.20–3.45)
TT cellularity 2 *vs*. 0	0.24 (0.05–1.07)
Tumor location left *vs*. right	7.90 (2.41–25.87) *
Open *versus* laparoscopic colectomy	16.73 (2.78–100.69)*
Anastomotic leakage	0.03 (0.005–0.24)*

TT: tumoral tissue, NT: normal tissue, CI: Confidence Intervalle.

* statistically significant.

#### Preanalytical parameters

The mean cold ischemia time was 70.2 ±50.9 minutes and median 60 minutes [5–325]. The cold ischemia time was not statistically different between the RQI <5 (59.9 minutes ±44.4) and RQI ≥5 groups (73.6 minutes ±52.5). As in normal tissue, neither the sample weight, nor the storage duration was statistically different between the two groups.

#### Histological and molecular parameters

Adenocarcinoma not otherwise specified was the most common adenocarcinoma type (n = 206), followed by mucinous adenocarcinoma (n = 25), serrated adenocarcinoma (n = 5), and micropapillary adenocarcinoma (n = 1).

RQI results were not influenced by adenocarcinoma subtype or by the presence or percentage of a mucinous component. There was no statistically significant difference between RQI <5 and ≥5 groups for tumor grade, cellularity, ulceration and necrosis. Malignant to stromal cells ratio had an influence on RQI value. Indeed, samples with RQI ≥5 had a superior stromal cell proportion (mean of +8.7%; p <0.05). However, RQI was not influenced by the percentage of stromal inflammatory cells (p = 0.95), endothelial cells (p = 0.41) and fibroblasts (p = 0.86). There was no statistically significant difference in MSI, *BRAF* and *KRAS* status between the two RQI groups.

Histological control revealed that 4 samples (1.7%) were not adenocarcinomatous but adenomatous remnants. In 15 cases (6.2%), non-tumoral colonic tissue involved almost the entire sample with less than 5% of tumoral tissue. Thus, at least 7.9% of the samples were not appropriate for molecular analyses because of tissue sampling inadequacy. The percentage of malignant cells was >60% in 66 cases (27.3%). Among these cases, necrosis involved >20% of the sample surface in 7 cases. In total, only 59 cases among 241 (24.4%) were considered histologically appropriate for molecular analyses after histological control according to the Cancer Genome Atlas criteria (TGCA) [33].

Immunohistochemical expression of HIF-1α and cleaved caspase 3 in stromal and tumor cells did not reveal significant difference between the RQI <5 and ≥5 groups.

#### 5’3’ ratio mRNA integrity assay and RQI values

**Fig 2:**
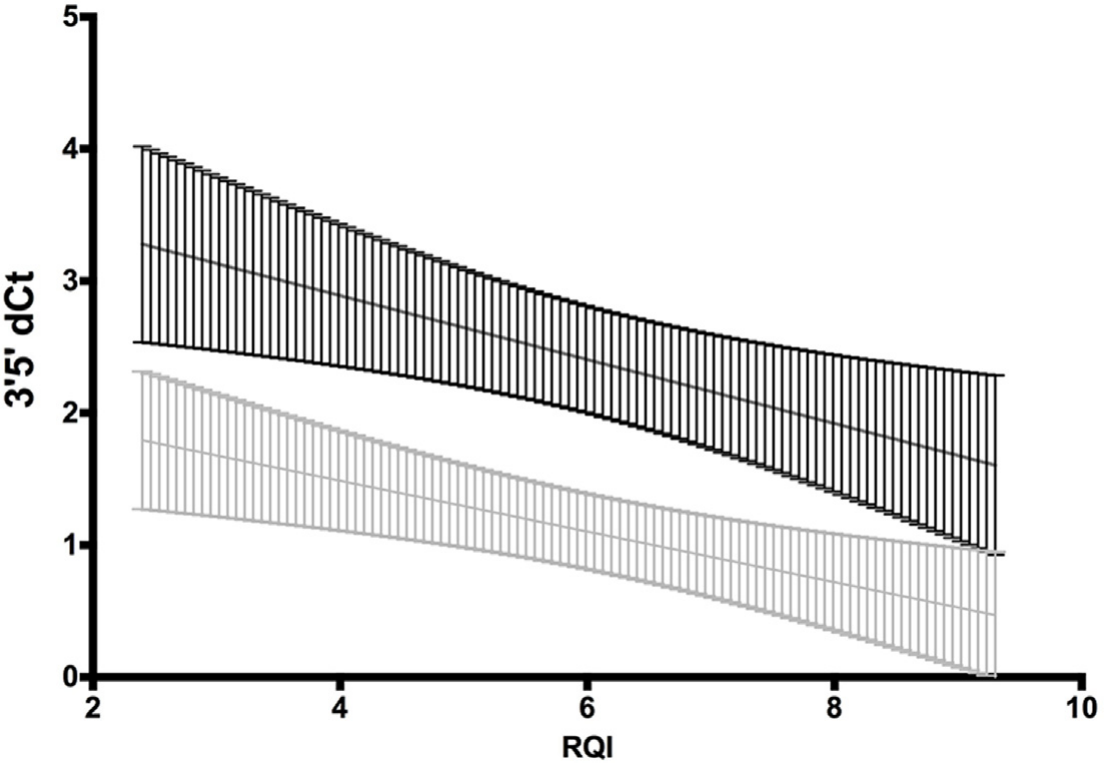
3’5’ dCt ratio of 2 housekeeping genes versus RNA RQI values. 3’5’ dCt RT-qPCR ratio of *TBP* (black) and *B2M* (gray) housekeeping gene measured in adenocarcinomatous samples. The ratio is inversely correlated to the RNA integrity assessed by the RQI.

To demonstrate the accuracy of RQI, 12 RNA samples with various RQI between 2.4 and 9.3 were subjected to 5’3’ ratio mRNA integrity assay and qPCR analysed for *TBP* and *B2M* genes. We found a clear increase in the 5’3’ dCt ratio correlated with the RNA integrity comparatively to the high quality RNA samples. These results are presented in Fig 2 and in S1 Fig. Moreover, we calculated 3’/5’ ratio using the mean 3’ relative expression value in optimal-quality samples (a 1 ratio indicated a non degraded RNA). For RNA with RQI>5 we found a mean 2.89 3’/5’ ratio for B2M and 1.36 for TBP. These ratios were 5.01 and 3.18 for RNA with RQI<5 for B2M and TBP respectively. They were in accordance with the quality criteria proposed by Die et al [34]. Indeed values near or below the limit of 2.73 for a 1100pb distance between 5’ and 3’ amplicons (1482pb for TBP in our experiments) and 1,36 for 700pb (813pb for B2M in our experiments) characterise cDNA as reliable for gene expression studies.

## Discussion

Clinical studies and medical research projects become more and more dependent on human tissue of high quality collected in Biobanks [35]. Knowledge of parameters influencing the RNA integrity of colonic biospecimens is a necessary prerequisite to improve the quality of biospecimens and, by extension, the molecular results. In the present study we evaluated the influence of different clinical, histological, and molecular parameters on the RQI results.

RNA degradation depends on tissue type [17]. Frozen samples from the gastrointestinal tract, especially pancreatic, gastric and colorectal samples, have lower RNA quality when compared to samples from other organ sites such as breast, tonsil, kidney, prostate, etc. [17,26]. Within the colonic samples, we found, as in previous studies, that RNA quality was higher in tumor tissues than in normal mucosa whatever the localization [36,37]. In our study, the location of the tumor was the 1 of the 3 parameters that significantly influenced RQI values on multivariate analysis. The influence of the location of colonic adenocarcinoma on RNA quality had not been described before. The difference in enzymatic environment and colonic bacteria flora between right and left colon might partly explain these results [38]. Previously, it was shown that the RNA from human and rat mucosal tissue of the proximal small intestine degrades much faster than the RNA of distal small intestine, caecum or colon with rectum [38]. This suggests a possible impact of enzymatic and bacterial environment on RNA quality within the intestinal tract. However, in our study RQI from right and left normal colonic mucosa were not statistically different. Thus, this right/left RQI difference is specific to the tumor. It is well known that right colonic adenocarcinomas have distinct molecular characteristics with more frequent microsatellite instability and *BRAF* mutation compared to left colonic adenocarcinomas [1]. In our study, *BRAF* and MSI status did not significantly influence RQI results. Thus, the right/left RQI difference in tumor tissue is not related to these genotypes.

In addition to the tumor location, in our study the type of surgical approach and the occurrence of post-surgery complications such as anastomosis leakage significantly influenced RQI results of tumor samples only on multivariate analysis. In our study, RNA quality of the tumor samples was lower when a laparoscopic colectomy was performed or when an anastomotic leakage occurred. We are the first to describe such relation between RNA quality and surgical approach or suture failure.

In one previous study, the RNA transcript of several genes decreased in case of laparoscopic colectomy compared to open colectomy [39]. In other studies, mRNA expression of some protease such as metalloproteinase 2 and 9 and urokinase-type plasminogen activator increased in colon carcinoma cell lines when submitted to CO_2_ concentration corresponding to laparoscopy conditions [40,41]. This increase of protease transcription related to high CO_2_ concentration cannot explain the decrease of RNA quality in tumor tissue. However, the CO_2_ pneumoperitoneum induces changes in gene expression of colonic tumor cells.

Anastomotic leakage is a rare but very severe complication of colon cancer surgery. Male patients, elderly patients, patients with comorbidity, patients with previous abdominal surgery, with pre-operative or intra-operative septic condition, with post-operative blood transfusion and patients who underwent right hemicolectomy are at higher risk of anastomosis leakage after colectomy [42–44]. The reason for the lower quality of tumor RNA in case of post-surgery anastomotic leakage remains unexplained but could be related to patients’ medical condition and to tumor location.

Despite rigorous tissue banking process, only 82.2% of frozen colonic tumoral samples had a RQI ≥5, and 57.3% had a RQI ≥7, whereas 94% had a RIN ≥7 in one previous study [36]. This difference might partly be explained by some preanalytical factors such as warm ischemia, surgical procedure, transport procedure, tissue manipulation, sampling or RNA extraction procedure. In our study, the sample weight had no impact on RQI. To our knowledge, this parameter had not been evaluated before.

As in previous studies [19,24,36,45], we found that storage duration of colonic biospecimens did not impact on RNA quality.

Our results showed that RNA quality was not significantly affected by cold ischemia time. These results are consistent with previous results on colonic biospecimens. In one study RNA integrity did not show decrease with cold ischemia for up to 4 hours [36]. Another study on fresh normal colon tissue left at room temperature, or kept on ice, or in saline or in RNAlater during 0.5, 1, 3, 6 and 16 h showed that RNA integrity remained stable under all conditions tested for up to 6–16 h [18]. Similar results were found on other organs such as pancreas [24] or tonsil [18]. In contrast, several other studies reported a progressive deterioration in RNA integrity on colonic tumor samples with increasing cold ischemia time up to 2h [46,47].

Although RNA degradation measurement is an objective evaluation of RNA quality, the direct effect of RNA degradation on gene expression levels must also be considered. In fact, the variations of housekeeping genes Ct with RQI are problematic because the accuracy of the results obtained by quantitative RT-PCR strongly depends on accurate transcript normalization using stably expressed housekeeping genes [48]. In our study, we observed that 5’3’dCt ratios of 2 commonly used housekeeping genes (*TBP* and *B2M*) were inversely proportional to RQI and 3’/5’ ratios indicated that oligodT priming cDNA are suitable for gene studies if RNA RQI is over 5. Thus, for RT-qPCR studies using colon cancer tissues, samples with high quality RNA (RQI >5) should preferably be used. Gene expression profiling studies on colorectal cancer showed that mRNA expression changes with handling time [36, 49] or RNA quality [31] concerned only few mRNA and would not be expected to play a major role in gene expression-based tumor stratification [37]. The genes that show the strongest effect due to RNA degradation were those with short mRNA and probe position near the 5’ end. It was concluded that degraded RNA from tumor samples could be still used to perform gene expression analyses [31]. Moreover, the impact of variation in RNA quality on estimate of gene expression levels can be corrected [50].

Histomorphological control of the biobanked specimens is crucial to ensure that the biospecimens were properly sampled. In our study, almost 8% of the tumoral biospecimens did not concern tumor tissue but the surrounding adenomatous or normal tissues. Similar results were found in previous studies where 10% to 25% of the frozen samples were unsuitable for a molecular analysis because of tissue sampling inadequacy [29,46].

Histological evaluation of the biospecimens also aims to estimate the malignant cell percentage. The suitable threshold of malignant cells percentage for gene expression analyses on colonic samples is actually unknown. However, in most studies and particularly in TGCA studies samples with more than 60% of neoplastic cells were used to ensure that the gene expression results reflect the tumor cell results and not the stromal cells results [11,28,34,36,46,51,52]. In our study, only 27.3% of the cases had neoplastic cells content >60% and thus considered as appropriate for molecular analyses according to the literature [12]. In other studies, 64.3 to 90% of the samples fulfilled this criterion [31,46,52]. This significant difference between studies may be explained by the lack of reproducibility of cell percentage estimation between pathologists [53]. The ratio of malignant cells nuclei to all other nuclei in the tissue is the only criterion that should be assessed for tumor cells percentage estimation [12]. Centralizing histological analysis may help to reduce the variability in malignant cell percentage and other histological criteria evaluation [28].

Regarding the influence of tumor cell percentage on RNA quality, we found better RQI in samples with lower malignant/stromal cell ratio. These results could be explained by the role of stromal cells to maintain a kind of homeostasis to prevent RNA degradation.

Another histological parameter usually evaluated on biospecimens is the necrosis extent. The maximal necrosis extend threshold is 20% in TGCA studies [12,28,33]. However, in our study as in other studies necrosis extent had no significant influence on RNA quality [12]. Because the necrosis extent estimation is not yet standardized and had no demonstrated impact on RNA quality, its importance on biospecimens quality evaluation should be reconsidered. Moreover the type of necrosis (coagulative or inflammatory) seems to be more important to specify [12].

In our study, the adenocarcinoma subtype, the cellularity and the presence of a mucinous component did not significantly influence RNA quality. In the study of Kap et al. a low cellularity of tumor sample was correlated with lower RIN results [19]. However, as necrosis extent evaluation, this morphological criterion is not well defined and is difficult to evaluate reproducibly.

Morphological control can be performed by 3 different techniques: 1/ on frozen tissue itself, 2/ on imprint cytology slide and 3/ on mirror slide. These 3 techniques have their own advantages and disadvantages [29]. The mirror image control consists in sampling a tissue close to the frozen sample and to perform a conventional histological analysis. This technique gives a very good morphological aspect of the tissue allowing immunohistochemical studies; however it analyses the tissue next to the frozen sample and not the frozen tissue itself. The correlation of frozen tissue/mirror tissue histology has been reported to be excellent [54,55].

We were the first to evaluate the relationship between ischemia and apoptosis marker (HIF-1α and cleaved caspase 3 respectively) immunohistochemical expression and RNA integrity. Our results showed no statistical difference in HIF-1α and cleaved caspase 3 immunohistochemical expression between RQI <5 and ≥5 groups. Thus, these 2 immunolabelling cannot be used as RNA quality indicator on mirror samples. In previous studies, variation of HIF-1 α gene expression and immunoreactive score with ischemia duration was controversial [49,56]. This lack of expected association between HIF-1α immunoreactive score and ischemia time could be explained by oncogene activation of HIF-1α in cancer [56,57].

In conclusion, we found that RNA quality is lower in normal colonic tissue compared to malignant colonic tissue, whatever the location. In colonic tumor samples only, RNA quality is influenced by some clinical and surgical parameters such as tumor location, type of surgical approach and the occurrence of suture failure. Further investigations are needed to explain these observations.

In our study, RNA quality has no relationship with preanalytical, morphological or molecular parameters.

For the meantime, these results suggest that tumor sampling and interpretation of molecular results should be performed more carefully for tumor tissue from laparoscopic colectomy specimens and right-sided tumors.

## Supporting Information

S1 Fig 3’ and 5’B2M housekeeping gene amplification compared to RQI. 3’ and 5’B2M housekeeping gene amplification by PCR in exponential phase (21 cycles) using 3’ and 5’B2M primers respectively after oligodT primed reverse transcription (top panel) and quantification by densitometry of the 3’5’ ratio (%3’5’) according to the RQI of RNA (lower panel). RNA with a 9.1 RQI was used as positive control. mRNA integrity is definitively correlated to RQI level, and RQI<5 is not suitable for RT-qPCR analysis.
(TIFF)
