# Supplementary figures and images for: Biobanking of Fresh-Frozen Human Adenocarcinomatous and Normal Colon Tissues: Which Parameters Influence RNA Quality?
Source: PLoS One. 2016 Apr 28;11(4):e0154326. doi: 10.1371/journal.pone.0154326 (PMC4849710; doi:10.1371/journal.pone.0154326)

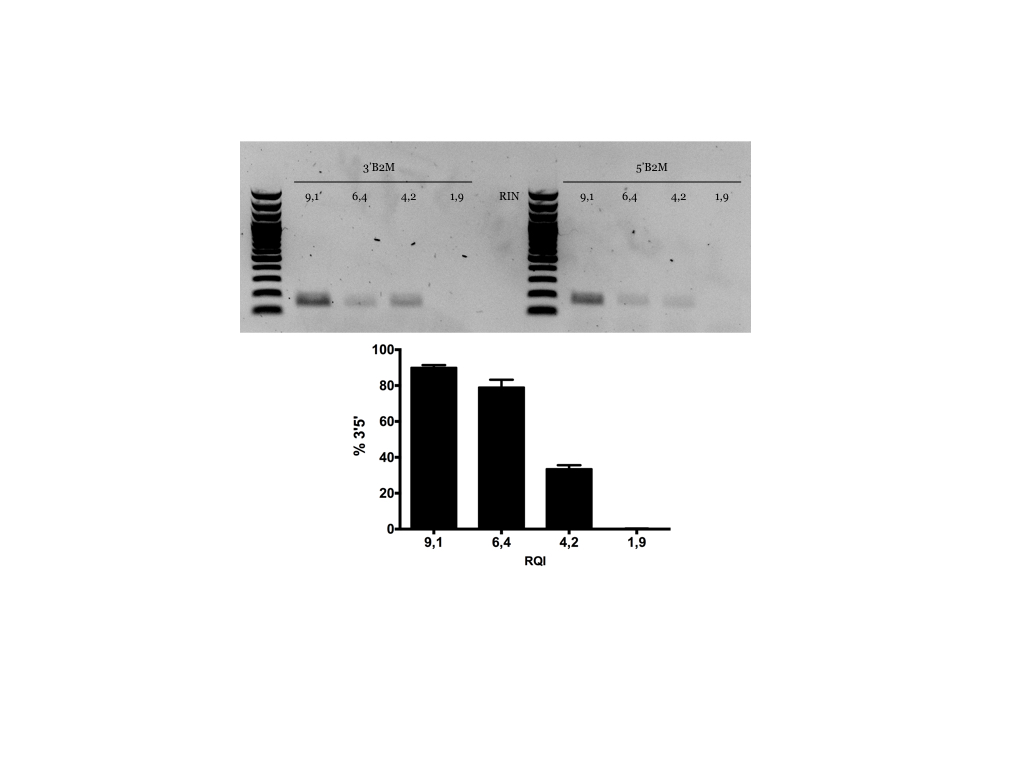

Supplement: S1 Fig — 3’ and 5’B2M housekeeping gene amplification by PCR in exponential phase (21 cycles) using 3’ and 5’B2M primers respectively after oligodT primed reverse transcription (top panel) and quantification by densitometry of the 3’5’ ratio (%3’5’) according to the RQI of RNA (lower panel). RNA with a 9.1 RQI was used as positive control. mRNA integrity is definitively correlated to RQI level, and RQI<5 is not suitable for RT-qPCR analysis. (TIFF) [file pone.0154326.s001.tiff]
